# T-cell redirecting therapies in lung cancer - a comprehensive analysis of clinical trials

**DOI:** 10.3389/fimmu.2026.1917770

**Published:** 2026-09-03

**Authors:** Jiahe Zhao, Ayrton Bangolo, Lili Zhang, Martin Gutierrez, Miguel Gonzalez-Velez

**Affiliations:** 1 Department of Hematology and Oncology, John Theurer Cancer Center, Hackensack University Medical Center, Hackensack, NJ, United States; 2 Department of Thoracic Oncology and Phase I Therapeutics, John Theurer Cancer Center, Hackensack University Medical Centerr, Hackensack, NJ, United States

**Keywords:** bispecific T cell engager, chimeric antigen receptor T (CAR T) therapy, lung cancer, T-cell redirected therapy, TCR-engineered T cells, tumor-infiltrating lymphocyte

## Abstract

Lung cancer remains the leading cause of cancer-related mortality worldwide, and despite the transformative impact of immune checkpoint inhibitors (ICIs), primary and acquired resistance, limited efficacy in immune-cold tumors such as extensive-stage small cell lung cancer (ES-SCLC), and the absence of durable responses in a substantial proportion of patients define the unmet need that T cell-redirected therapies aim to address. These strategies engage, engineer, and deliver cytotoxic T cells against tumor cells independently of preexisting immunity, and this review provides a comprehensive overview of the four major modalities under clinical development in lung cancer: bispecific T cell engagers (BiTEs), tumor-infiltrating lymphocyte (TIL) therapy, chimeric antigen receptor T cell (CAR-T) therapy, and T cell receptor-engineered T cell (TCR-T) therapy. Among these, tarlatamab, a DLL3×CD3 BiTE, has achieved the most advanced clinical validation, receiving traditional FDA approval in November 2025 for second-line ES-SCLC following Phase 3 DeLLphi-304 data demonstrating superior overall survival versus chemotherapy (median 13.6 vs. 8.3 months; HR 0.60). TIL therapy is the leading adoptive cell approach in non-small cell lung cancer (NSCLC), with lifileucel demonstrating a 25.6% ORR in anti-PD-1-resistant disease and a 64.3% ORR when combined with pembrolizumab in ICI-naïve patients, though manufacturing complexity and cost constrain broad access. CAR-T and TCR-T therapies remain in early proof-of-concept phases, limited by the absence of uniformly expressed tumor-restricted surface antigens in NSCLC, MHC class I downregulation, and the sub-1% eligibility rates imposed by HLA restriction in TCR-T programs, while generating important engineering insights around genome editing, armored cytokine payloads, and neoantigen targeting strategies. Shared challenges across all modalities include antigen loss under therapeutic pressure, T cell exhaustion driven by the immunosuppressive lung tumor microenvironment, and the absence of prospectively validated predictive biomarkers for patient selection. Combination strategies pairing T cell-redirected therapies with ICIs, antibody-drug conjugates, and allogeneic or innate immune effectors represent the most promising path toward durable benefit. As the field advances toward Phase 3 trials, biomarker-selected patient populations, and scalable allogeneic manufacturing platforms, T cell-redirected therapy is positioned to extend the survival frontier for patients whose lung cancer has escaped existing treatments.

## 1 Introduction

Lung cancer is the leading cause of cancer-related mortality worldwide. According to estimates by GLOBOCAN 2022, it accounts for approximately 2.5 million new diagnoses of cancer annually worldwide, which represents around 12.4% of all cancer cases, and 1.8 million deaths, or 18.7% of all cancer deaths (1). It accounts for more cancer deaths than any other malignancy, and it is the leading cause of cancer death among men across the majority of world regions. With the global projections forecasting a rise to 35 million new cancer cases annually by 2050, that would increase the burden of lung cancer substantially in the coming decades (1).

Despite declining tobacco use in many countries, mostly in high-income countries, incidence continues to rise (1, 2). This reflects ongoing tobacco use in low and middle-income countries, an aging population, and a growing burden of lung cancer in never-smokers driven by outdoor air pollution (PM2.5), indoor biomass combustion, radon, and occupational exposures (2, 3). If lung cancer in never-smokers is considered independently, it is the fifth leading cause of cancer mortality (2). Air pollution accounts for approximately 14.1% of lung cancer deaths globally, with cases occurring disproportionately in Asia (3), underscoring that non-tobacco factors are becoming increasingly central to the disease’s epidemiological trajectory. This growing and increasingly heterogeneous burden falls on a treatment landscape that, despite a decade of immunotherapy, still leaves most patients without durable benefit.

Since the introduction of anti-PD-1/PD-L1 immune checkpoint inhibitors (ICIs), they have fundamentally changed the treatment landscape of lung cancer. Medications such as pembrolizumab and nivolumab are cornerstones of therapy and substantially extend survival in a significant subset of patients with advanced non-small cell lung cancer (NSCLC) (4–7). Despite this, the clinical benefits of ICIs have remained inconsistent, and non-durable for many patients. Primary resistance affects a substantial proportion of patients, and acquired resistance remains another significant factor limiting long-term effectiveness through mechanisms such as loss of antigen presentation, tumor microenvironment factors, and clonal evolution (8–10).

In small cell lung cancer (SCLC), the addition of checkpoint inhibitors to first-line chemotherapy produced statistically significant but modest survival gains in IMpower133 and CASPIAN trials, improvements of roughly two to three months in median overall survival (hazard ratio 0.70 and 0.73, respectively), a benefit sufficient to establish chemoimmunotherapy as the first-line standard of care in ES-SCLC, without durable immunological control for most patients, which is attributed to the predominantly immune-cold nature of this entity (11–14). T cell-redirected therapies represent a mechanistically distinct response to these limitations, engaging cytotoxic T cells directly against tumor cells independently of preexisting immunity, and the field has now moved from early investigation to regulatory validation, exemplified by the 2025 FDA traditional approval of tarlatamab-dlle for second-line for ES-SCLC (15).

T cell redirection is particularly relevant in cases where the immune response is absent, exhausted, or excluded by mechanisms the tumor can use to acquire resistance. There are four major modalities under clinical investigation right now for lung cancer in this new field: bispecific T cell engagers (BiTEs), which bridge T cells and tumor cells via tumor antigen and CD3 binding; CAR-T cells therapy, which engineers T cells ex vivo to express tumor-targeting receptors that are MHC-independent; TCR-engineered T cell (TCR-T) therapy, which redirects T cells with introduced TCRs to access intracellular antigens via peptide-HLA presentation; and TIL therapy, which harvests the patient’s own tumor-reactive T cells, expands them and reinfuses them (10, 15, 16).

These are the immunological gaps that T cell redirection is designed to close, and they frame the scope of this review, which provides a comprehensive overview of the current landscape of T cell-redirected therapy across both NSCLC and SCLC. We examine the rationale for using T cell redirections and their immunological foundations, the clinical evidence and safety of these modalities, including tarlatamab for SCLC, for which FDA approval has been achieved, and the current trials underway studying new medications or in combination with established therapies. We also examine the shared mechanisms of resistance across modalities and the toxicity management frameworks currently in clinical use. We conclude with the future directions that will shape the integration of these therapies into current clinical management of lung cancer, and with how patient selection can determine the scenarios in which they would be more beneficial.

## 2 The immunological barrier in lung cancer

### 2.1 What makes the lung tumor microenvironment distinct

The immune microenvironment of lung cancer is highly variable, and this variability is a central determinant of whether T cells can recognize and destroy tumor cells. In NSCLC, tumors span a wide immunological spectrum, from immunologically “hot” tumors with substantial T cell infiltration to “cold” or “excluded” tumors, where immune cells are physically prevented from reaching the tumor core by stromal barriers and a network of immunosuppressive signals, including those from tumor-associated macrophages and inhibitory cytokines, that collectively block effective antitumor immunity (17, 18). SCLC presents as a more hostile landscape, it arises from neuroendocrine cells, its tumors are predominantly immune-cold, characterized by sparse T cell infiltration, low surface expression of the molecules required for antigen presentation, and high expression of immunosuppressive ligands (11, 14, 19). Molecular subtyping of SCLC has identified four transcription factor-defined subtypes (SCLC-A, -N, -P, and -I), each with distinct molecular vulnerabilities, of which only the SCLC-I subtype carries an inflamed gene expression signature and derives the greatest benefit from the addition of immunotherapy to chemotherapy, while the remaining three subtypes are predominantly immune-cold and show preferential sensitivity to inhibitors of PARP, Aurora kinases, and BCL-2, respectively, underscoring that immune-cold biology is the dominant but not universal feature of the disease (20). Across both histologies, the microenvironment limits the effectiveness of endogenous antitumor immunity and underlies the incomplete and inconsistent responses seen with immune checkpoint inhibitors, establishing the fundamental rationale for T cell-redirected strategies that deliver or engage cytotoxic T cells directly at the tumor, regardless of the underlying immunological state.

One axis in this environment is both specific to the lung and self-reinforcing. Hypoxia within the tumor stabilizes HIF-1α, which upregulates the adenosinergic machinery, CD39, CD73 and the adenosine receptors, while the same hypoxic conditions drive release of extracellular ATP that these ectonucleotidases convert into adenosine (21). The resulting adenosine acts broadly rather than on T cells alone, inhibiting effector T cells, NK cells, dendritic cells, macrophages and neutrophils, and promoting both T cell anergy and the differentiation of CD4+ cells toward Foxp3+ regulatory T cells (21). This is not a generic property of solid tumors. CD73 expression is unusually robust in NSCLC relative to other cancer types and is most pronounced in tumors driven by mutant EGFR and KRAS, which makes adenosinergic suppression a driver-linked feature of lung cancer rather than an incidental one (21). The myeloid compartment then feeds the same axis, since TGF-β drives myeloid-derived suppressor cells toward a terminally differentiated phenotype characterized by high surface CD39 and CD73, so that the suppressive cells accumulating in the tumor become adenosine producers themselves (21). Any redirected T cell delivered into a lung tumor enters this environment, and several of the engineering strategies discussed in Section 5.4, A2AR disruption most directly, are best understood as responses to it.

### 2.2 T cell dysfunction and immune evasion: implications for T cell-redirected therapy

When T cells are present within lung tumors, they frequently fail to eliminate malignant cells because of a progressive state of dysfunction driven by chronic antigen exposure and the suppressive tumor environment. This process, known as T cell exhaustion, results in the gradual loss of cytolytic function and the upregulation of inhibitory surface receptors, rendering T cells increasingly unable to kill their targets (22, 23). In its later stages, exhaustion becomes epigenetically fixed and largely irreversible, explaining why checkpoint inhibitors, which work by reinvigorating existing T cells, have limited efficacy once this terminal dysfunctional state has been established. Strategies that redirect or deliver cytotoxic T cells *de novo*, rather than attempting to rescue already exhausted populations, are better positioned to overcome this barrier (24).

Beyond T cell exhaustion, lung tumors actively evade killing through mechanisms that constrain both endogenous and redirected immune responses. Many tumors downregulate or lose the surface molecules responsible for antigen presentation such as MHC class I, effectively hiding from T cells that depend on this pathway for target recognition (16, 25, 26). This vulnerability applies specifically to TCR-dependent modalities such as TIL therapy and TCR-T cell therapy; BiTEs and CAR-T cells, which recognize surface antigens directly without MHC involvement, retain activity when antigen presentation is impaired. An additional suppressive mechanism involves the accumulation of adenosine within the tumor microenvironment, which broadly dampens T cell activation and cytotoxic activity in lung cancer (21). Exclusion, exhaustion, and active immune evasion define the immunological barriers that T cell-redirected therapy must overcome, and underpin the rationale for each modality discussed in the sections that follow.

## 3 Bispecific T-cell engagers

### 3.1 Mechanism of action and DLL3 as the defining target in lung cancer

**Figure 1:**
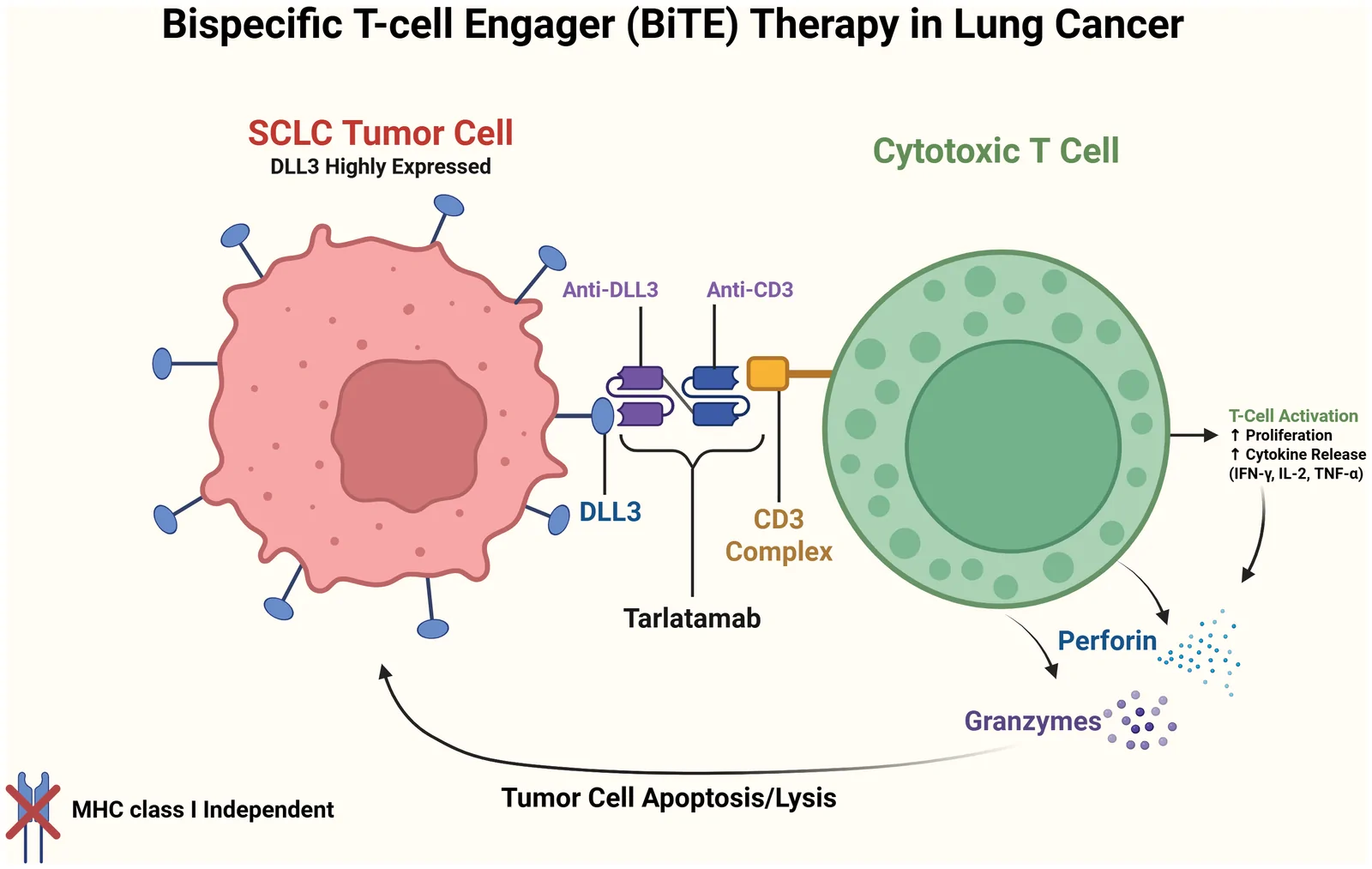
Mechanism of action of bispecific T-cell engager (BiTE) therapy in lung cancer. Tarlatamab simultaneously binds DLL3 on the SCLC tumor cell surface and the CD3 complex on a cytotoxic T cell, forming a cytolytic immunological synapse independent of MHC class I. This bridging triggers T-cell activation, proliferation, and cytokine release (IFN-γ, IL-2, TNF-α), leading to perforin- and granzyme-mediated tumor cell apoptosis/lysis. Created in BioRender. Zhang, L. (2026) https://BioRender.com/iq380xe.

Bispecific T-cell engagers are engineered antibody constructs that simultaneously bind a tumor-associated antigen on malignant cells and the CD3 complex on T cells, physically bridging the two to form a cytolytic immunological synapse that triggers T cell activation and tumor cell killing independently of MHC class I expression (**Figure 1**), enabling any T cell to kill any antigen-expressing tumor cell regardless of its intrinsic specificity (16, 27). Blinatumomab, the first BiTE to demonstrate tumor regression in cancer patients, established the clinical proof of concept for the class; classic BiTEs are small Fc-free molecules of approximately 55 kDa, a structure that facilitates deep tissue penetration but produces a short serum half-life necessitating continuous infusion, a limitation addressed by half-life extended (HLE) formats that incorporate an effector-functionless Fc domain to enable intermittent dosing, while next-generation architectures such as multi-valent, trispecific, and T cell receptor-mimic designs further expand potency, pharmacokinetic profiles, and the range of targetable antigens (16, 27–30). In small cell lung cancer, BiTE development has been defined almost entirely by delta-like ligand 3 (DLL3), a surface protein aberrantly overexpressed on more than 80% of SCLC tumors with minimal expression on most normal adult tissues, a therapeutic window first established through DLL3-targeted antibody-drug conjugate (ADC) studies and the Phase 1 evaluation of rovalpituzumab tesirine; although this ADC was ultimately discontinued after demonstrating inferior overall survival compared with topotecan in the Phase 3 TAHOE trial, it validated DLL3 as a target of interest and laid the groundwork for BiTE development (31–35). Critically, rovalpituzumab tesirine’s toxicity was attributable to its cytotoxic payload rather than to DLL3 itself: dose-limiting thrombocytopenia, serosal effusions and capillary leak syndrome, and photosensitivity skin reactions occurred independently of tumor DLL3 expression level and drove treatment discontinuation in 22% of patients (33), a payload-driven toxicity profile that T cell engagers do not share. The two modalities also differ in what they demand of the target. An antibody-drug conjugate must be internalized and release its payload intracellularly in quantities sufficient to kill the cell, so its activity scales with antigen density: rovalpituzumab tesirine produced an objective response rate of 18% overall but approximately 38% in DLL3-high tumors (33). A T cell engager needs only to nucleate a cytolytic synapse, and tarlatamab retains potent cytotoxicity against SCLC cells expressing very low DLL3, with DLL3-directed engagers producing responses across the range of expression levels (36). The same degree of DLL3 expression can therefore be insufficient for the conjugate and sufficient for the engager, which is why DLL3 status functions largely as an eligibility criterion for the engager but as a determinant of response for the conjugate. This difference is complementary to, rather than a substitute for, the toxicity explanation above, since the TAHOE trial enrolled DLL3-high patients specifically and the conjugate still underperformed topotecan, indicating that adequate target density could not compensate for a narrow therapeutic index (35).

### 3.2 Tarlatamab: clinical development, regulatory approval, and combination strategies

Tarlatamab, known as AMG 757 during its development, is a half-life extended DLL3×CD3 BiTE designed to optimize potency across the full spectrum of DLL3 surface expression while supporting biweekly administration; preclinical studies confirmed potent DLL3-specific T cell-mediated killing in SCLC cell lines and patient-derived xenograft models with a pharmacokinetic profile supporting clinical development (34). In the Phase 1 DeLLphi-300 trial, the first clinical study of tarlatamab in relapsed or refractory SCLC, doses ranging from 0.003 to 100 mg were evaluated across 107 patients, the maximum tolerated dose was not reached; 10 mg administered every two weeks was subsequently selected as the preferred dose following Phase 2 comparison with 100 mg, showing superior tolerability with maintained efficacy (37, 38). Tarlatamab demonstrated manageable safety, with cytokine release syndrome (CRS) occurring in 52% of patients but predominantly grade 1 or 2, transient, and largely confined to the first cycle, and mostly mild neurologic events occurring in 70%, with grade 3 or higher in 11.2% and discontinuation required in only 2 patients, the ORR was 23.4% with a median duration of response of 12.3 months, exceeding outcomes compared to standard second-line agents. Extended follow-up of patients receiving the 10 mg dose confirmed an ORR of 35.3% and a median overall survival of 20.3 months; intracranial activity was also observed, with central nervous system (CNS) tumor shrinkage of 30% or more in 62.5% of patients with evaluable brain lesions and intracranial disease control in 87.5%, suggesting that tarlatamab may achieve intracranial control though the exact mechanism requires further study (38, 39).

The pivotal Phase 2 DeLLphi-301 trial confirmed a 40% ORR at the 10 mg dose in previously treated ES-SCLC, with 59% of responders across both evaluated doses maintaining response for at least six months; a dedicated brain metastasis subgroup analysis found a higher ORR of 45.3% in patients with treated stable brain metastases compared to 32.6% in those without, without additional grade 3 or higher neurological toxicity at the 10 mg dose, with intracranial activity proposed to involve activated T cell CNS infiltration rather than direct drug penetration, distinguishing tarlatamab from most systemic agents in its capacity for CNS disease control (37, 40–42), results that supported accelerated FDA approval in May 2024. The confirmatory Phase 3 DeLLphi-304 trial subsequently demonstrated improved overall survival versus chemotherapy (median 13.6 vs. 8.3 months; HR 0.60; P<0.001), with superior response rates and fewer treatment-related grade 3 or higher adverse events, leading to traditional FDA approval of tarlatamab in November 2025, the first T cell engager to receive traditional approval in lung cancer, establishing it as the new standard of care for second-line ES-SCLC (43–45). Early real-world data from patients treated outside clinical trial criteria show an ORR consistent with DeLLphi-301 (42.9%), but CRS occurred in 72.7% of patients and immune effector cell-associated neurotoxicity syndrome (ICANS) in 40.9%, rates meaningfully higher than those reported in trials, likely reflecting the inclusion of patients with untreated brain metastases and other comorbidities that would have been excluded from the pivotal studies (46).

Building on second-line approval, the DeLLphi-303 Phase 1b study evaluated tarlatamab combined with a PD-L1 inhibitor as first-line maintenance following platinum-based chemoimmunotherapy in ES-SCLC, demonstrating a manageable safety profile and a median overall survival of 25.3 months from maintenance initiation, exceeding historical benchmarks for PD-L1 inhibitor monotherapy maintenance, and informing the ongoing randomised Phase 3 DeLLphi-305 trial of tarlatamab plus durvalumab versus durvalumab alone (47–49). The toxicity profile is dominated by CRS occurring in 56% of patients, comparable to the 51% rate observed with tarlatamab monotherapy in DeLLphi-301, predominantly grade 1 or 2 in both settings, with early onset and rapid resolution managed through a mandatory step-up dosing strategy; ICANS occurred in 6% of patients, all grade 1 or 2, with a median time to onset of 9 days and median resolution of 3 days, with no treatment discontinuations attributable to either toxicity in DeLLphi-303, a favorable safety signal, though current prescribing information still mandates 22–24 hours of monitoring in an appropriate healthcare setting after Cycle 1 Day 1 and Day 8, with patients advised to remain within one hour of a healthcare facility for 48 hours following each dose; fully outpatient administration of the step-up doses remains under prospective evaluation rather than established practice. These data more directly indicate that the addition of a PD-L1 inhibitor does not worsen the immune-mediated toxicity burden of tarlatamab (37, 47).

### 3.3 Other DLL3-targeted T cell engagers

The clinical validation of tarlatamab has catalyzed the development of additional DLL3-directed T cell engagers, with obrixtamig (BI 764532) demonstrating an ORR of 23% with a median duration of response of 8.5 months across SCLC, extrapulmonary neuroendocrine carcinomas, and large-cell neuroendocrine carcinoma of the lung in a Phase 1 study enrolling 168 heavily pretreated patients, including a 70% ORR in the large-cell neuroendocrine subgroup at recommended step-up dosing regimens, with CRS in 57% predominantly low-grade, grade ≥3 in only 3%, ICANS in 9% including two fatal events at high-dose regimens that informed the step-up dosing optimization now carried forward into combination trials, and MTD not formally reached, with multiple combination trials now underway evaluating obrixtamig alongside ezabenlimab and first-line standard of care (50). Gocatamig (HPN328, MK-6070), a trispecific construct incorporating DLL3 targeting, albumin binding for pharmacokinetic extension, and anti-CD3, demonstrated dose-proportional pharmacokinetics and confirmed antitumor activity across neuroendocrine malignancies in Phase 1/2 evaluation, with a confirmed ORR of 46% in non-SCLC neuroendocrine neoplasms and 15% in neuroendocrine prostate cancer at current dosing, and is now being assessed in separate combination arms with durvalumab or ifinatamab deruxtecan in an ongoing Phase 1/2 trial in unresponsive SCLC, extending the potential reach of DLL3-directed therapy across the spectrum of high-grade neuroendocrine tumors (36, 51–53).

### 3.4 T cell engagers beyond DLL3: targeting NSCLC

In contrast to SCLC, no T cell engager has yet been approved for NSCLC, reflecting the absence of a surface antigen with the homogeneous tumor expression and restricted normal tissue distribution that DLL3 provides in SCLC (54, 55). B7-H3, broadly overexpressed across NSCLC with favorable tumor-to-normal tissue ratios, had been the most clinically advanced CD3-engaging target in this setting until its leading program was discontinued in 2025 without disclosed efficacy data (56). EGFR, overexpressed in a large proportion of NSCLC tumors but also present on normal epithelial tissues, carries an on-target toxicity risk that has prompted protease-activated designs engineered to switch on selectively within the tumor microenvironment, with at least one such EGFR × CD3 T cell engager now in Phase 1 evaluation (57). HER3, while clinically validated as an antibody-drug conjugate target through agents such as patritumab deruxtecan, has not yet yielded a dedicated T cell engager program. None of these targets has established a clinical efficacy signal sufficient to define a leading BiTE candidate in NSCLC, reflecting that identifying an antigen with a therapeutic window equivalent to DLL3 in SCLC remains the central unmet need for T cell engager development in non-small cell lung cancer (54, 55).

### 3.5 Resistance to T cell engagers

Despite durable responses in a subset of patients, most treated with tarlatamab ultimately progress through resistance operating at two levels. Tumor-intrinsic mechanisms include downregulation or loss of DLL3 under therapeutic pressure, driven by alterations in Notch pathway signaling, upregulation of inhibitory ligands such as PD-L1 on tumor cells in response to IFN-γ secreted by TCE-activated T cells, and activation of anti-apoptotic pathways involving SERPINB9, BCL2L1, and CFLAR that render tumor cells refractory to cytolytic signals even when the immunological synapse is intact (58, 59). Lineage plasticity represents a specific route to this DLL3 loss: because DLL3 is a direct transcriptional target of ASCL1, response to DLL3-directed T cell engagers is concentrated in ASCL1-high tumors, and one recognized mode of acquired resistance is therapeutic selection for a NEUROD1-high state with concomitant DLL3 downregulation, effectively a lineage switch away from the ASCL1-driven, DLL3-expressing state that these agents depend on. Resistant tumors can also show enrichment of regulatory and exhausted T cell programs alongside this antigen loss, indicating that antigen-intrinsic and T cell-extrinsic resistance routes can co-occur (58, 59). Tumor-extrinsic mechanisms involve progressive T cell exhaustion from sustained antigen engagement, characterized by upregulation of inhibitory receptors including PD-1 and TIM-3, and suppression of redirected T cells by regulatory T cells secreting TGF-β and IL-10 and myeloid-derived suppressor cells within the immunosuppressive SCLC microenvironment (58, 59). Strategies under investigation to counter resistance include checkpoint inhibitor co-administration to prevent T cell exhaustion, dual-antigen targeting to prevent single-antigen escape, and antibody-drug conjugate combinations to reduce antigen-loss escape variants through tumor debulking, with a clinical trial collaboration now evaluating zocilurtatug pelitecan, a DLL3-targeting ADC, in combination with tarlatamab, simultaneously delivering cytotoxic payload and redirecting T cells against the same target (58–60).

## 4 Tumor-infiltrating lymphocyte therapy

### 4.1 Biology and rationale

TILs within the NSCLC tumor microenvironment carry significant prognostic weight, with high CD8+ and CD4+ T cell infiltration associated with improved survival and elevated FOXP3+ Treg density associated with inferior outcomes in some but not all studies, providing a direct rationale for mobilizing this endogenous immune population therapeutically (61, 62). Within this infiltrate, only a fraction of T cells carry clones with specificity for mutation-associated neoantigens, but their cytolytic function is frequently impaired by the exhaustion programs described in Section 2.2, in the tissue-resident form characteristic of the lung (63). TIL therapy exploits this biology by harvesting tumor-reactive cells, expanding them ex vivo in a cytokine-rich environment free from immunosuppressive constraints, and reinfusing activated T cells, circumventing the requirement for endogenous T cell activation and bypassing the exhaustion that limits the efficacy of checkpoint inhibitors in established tumors (64–68).

### 4.2 TIL manufacturing: from tumor to product

**Figure 2:**
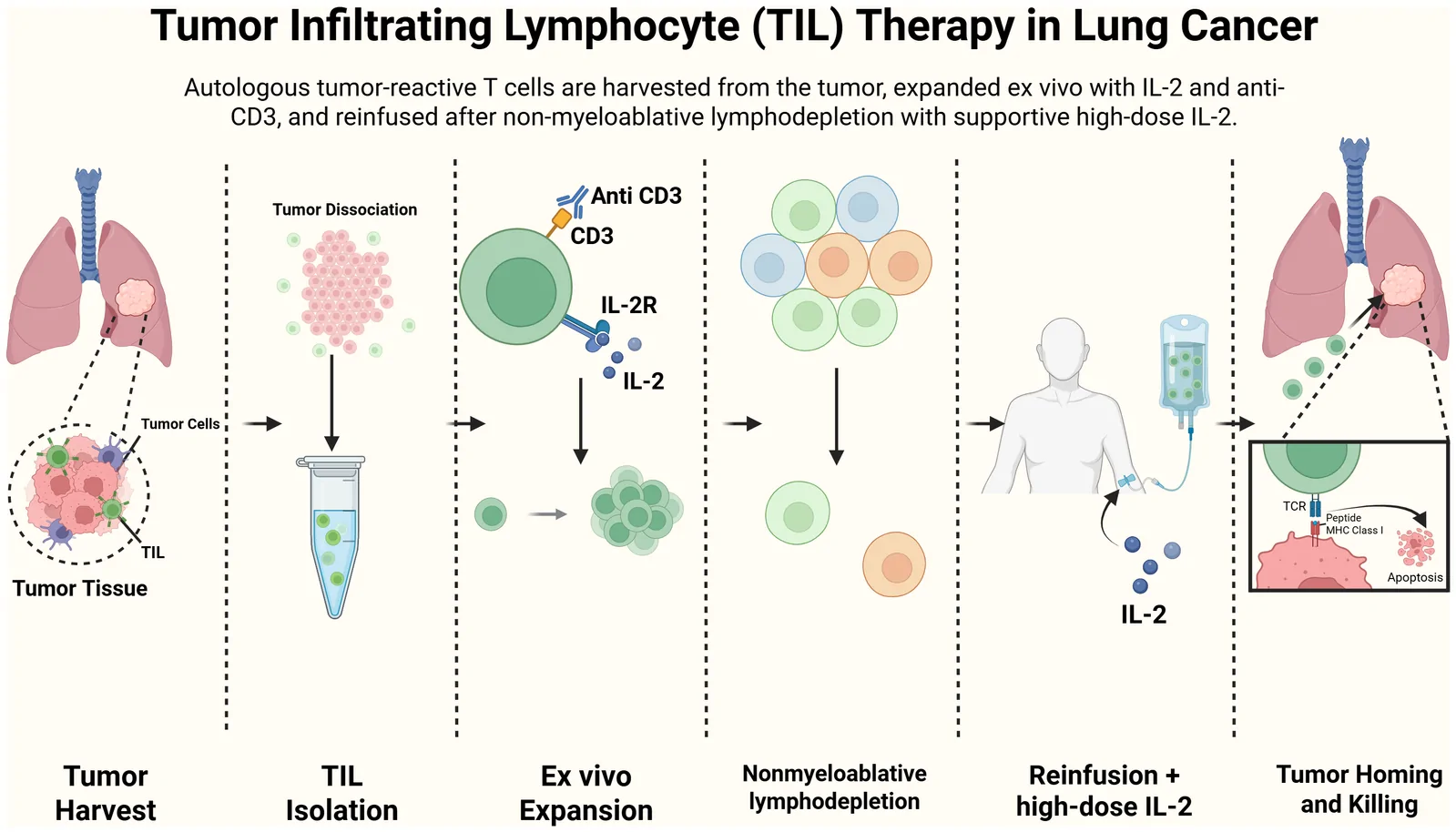
Manufacturing and delivery of tumor-infiltrating lymphocyte (TIL) therapy in lung cancer. Autologous tumor-reactive T cells are harvested from resected tumor tissue, isolated, and expanded ex vivo with IL-2 and anti-CD3 stimulation. Following non-myeloablative lymphodepletion, expanded TILs are reinfused together with supportive high-dose IL-2, enabling tumor homing and T cell-mediated tumor killing. Created in BioRender. Zhang, L. (2026) https://BioRender.com/63xtgal.

TIL manufacturing begins with tumor resection, followed by culture of tumor fragments with high-dose interleukin-2 (IL-2) to promote initial T cell outgrowth, and a rapid expansion protocol (REP) using feeder cells and anti-CD3 stimulation to generate the large cell numbers required for infusion (**Figure 2**) (66, 69, 70). Innovations such as the TIL 3.0 protocol, which replaces the conventional IL-2–only initial culture with combined IL-2, anti-CD3, and 4-1BB stimulation, improve cell yield and better preserve tumor-reactive clonotype hierarchy compared to the traditional TIL 1.0 method, with the subsequent REP itself identified as a further source of clonotypic drift that TIL 3.0 alone may ultimately circumvent (71). TIL 3.0 delivers all three signals required for optimal T cell activation, CD3 ligation, 4-1BB costimulation, and IL-2 exposure. In contrast to TIL 1.0’s reliance on IL-2 alone. In a head-to-head comparison using paired NSCLC tumor samples, this produced 5.3-fold more TIL per successful expansion, a higher overall success rate, and shorter manufacturing time than TIL 1.0, together with greater enrichment of CD8+ T cells and better preservation of the tumor’s original clonal hierarchy, including a higher proportion of the ten most prevalent tumor-infiltrating clones (71).

This process has been standardized into a centralized 22-day GMP manufacturing protocol (Gen-2 process) for lifileucel, with non-myeloablative lymphodepletion administered to patients before infusion to create immunological space, followed by high-dose IL-2 support to sustain *in vivo* T cell expansion (66, 72, 73).

### 4.3 Clinical evidence for TIL therapy in lung cancer

The C-144–01 Phase 2 trial established clinical proof of concept for lifileucel in heavily pre-treated patients with unresectable or metastatic melanoma who had received prior anti-PD-1/PD-L1 therapy and, if BRAF V600 mutation-positive, BRAF ± MEK inhibitors, with an objective response rate of 36%, a median duration of response not reached at 18.7 months of follow-up, and successful lifileucel infusion achieved in 66 of 78 patients who underwent tumor resection (74). FDA approval of lifileucel in February 2024 as the first cellular therapy approved for a solid tumor provided the regulatory foundation for extending TIL therapy to lung cancer and other epithelial malignancies (75, 76). The five-year follow-up of the C-144–01 melanoma study confirmed a median duration of response of 36.5 months, with responses continuing to deepen over time, including four conversions from partial to complete response more than one year after infusion, and 31.3% of responders maintaining ongoing responses at five years, with no additional long-term safety concerns (75). These findings provide evidence that memory-phenotype tumor-reactive T cells from the infused product can sustain durable immunosurveillance, a biological principle applicable to the ongoing NSCLC TIL programs. Prior to lung cancer-specific trials, a Phase 2 study of TIL therapy in HPV-associated epithelial malignancies demonstrated objective responses in 28% of patients with metastatic cervical cancer and 18% in other HPV-positive carcinomas, with two complete responses remaining ongoing more than four years after treatment, establishing proof of concept that TIL-mediated regression extends to epithelial cancers beyond melanoma (77).

The first clinical trial of TIL therapy in lung cancer enrolled 20 patients with advanced NSCLC who had progressed on nivolumab monotherapy, who then received TIL infusion with cyclophosphamide and fludarabine lymphodepletion and IL-2, followed by maintenance nivolumab. Among 13 evaluable patients, 3 achieved confirmed objective responses (ORR 23%), including 2 complete responses both ongoing at 1.5 years, and 11 of 13 had some reduction in tumor burden with a median best change of −35%. In exploratory analyses, T cells recognizing multiple cancer mutations were detectable after treatment, neoantigen-reactive clonotypes increased and persisted in peripheral blood, and these clonotypes were enriched in responding patients, providing direct mechanistic evidence of tumor-specific activity (64). An early-phase analysis of lifileucel in IOV-COM-202, a multicenter basket study, demonstrated an ORR of 21.4% (6/28) in previously treated mNSCLC, with responses in PD-L1-negative, TMB-low, and STK11-mutant tumors resistant to standard immunotherapy (78). The subsequent dedicated Phase 2 IOV-LUN-202 trial in non-squamous NSCLC reported an updated ORR of 25.6%, DCR of 71.8%, and a median duration of response not yet reached at 25.4 months (79, 80). An updated reduced lymphodepletion regimen introduced during the trial improved tolerability, cutting post-IL-2 hospitalization time by more than half and reducing cytopenia incidence without affecting efficacy (78–80).

The IOV-COM-202 Phase 2 study evaluated lifileucel combined with pembrolizumab in checkpoint inhibitor-naive advanced NSCLC, with updated results at a median follow-up of 25.6 months demonstrating an ORR of 64.3% in the EGFR-wild-type subgroup, including an ORR of 54.5% in patients with PD-L1-negative tumors, and durable ongoing responses at last follow-up (65, 81). These findings suggest that the polyclonal tumor-reactive TIL repertoire and PD-1 checkpoint disinhibition act cooperatively, with the TIL product broadening antigen coverage and pembrolizumab preserving the functional longevity of infused T cells within the immunosuppressive lung tumor microenvironment.

### 4.4 Challenges for TIL therapy in lung cancer

TIL therapy faces several interconnected challenges that must be addressed before broad clinical deployment in lung cancer is achievable. The vein-to-vein manufacturing timeline spans four to six weeks, demanding bridging therapy in rapidly progressing patients and specialized GMP infrastructure currently concentrated at academic centers; the cost of commercially manufactured lifileucel, exceeding $500,000 per treatment, creates access barriers, though hospital-based in-house manufacturing at approximately $55,000 per patient offers a potential alternative (66, 72). Patient fitness requirements are demanding: the non-myeloablative lymphodepletion regimen combined with high-dose IL-2 produces acute toxicity, including hypoxia, febrile neutropenia, and prolonged cytopenias, excluding patients with poor performance status or significant comorbidities, though modified reduced-intensity regimens have demonstrated promise in reducing this burden without compromising efficacy (65, 79, 81). Adequate tumor tissue is required to initiate manufacturing, and even when sufficient tissue is available, the majority of infused TILs are non-tumor-reactive T cells, diluting the therapeutic fraction and limiting cytolytic potency (63, 72). After infusion, TILs must traffic to tumor sites, penetrate dense stroma, and survive within an immunosuppressive microenvironment capable of driving re-exhaustion (82); manufacturing innovations that preserve less-differentiated T cell phenotypes, such as the TIL 3.0 protocol, aim to improve post-infusion persistence (71, 82). Patient selection for TIL therapy remains constrained by the absence of validated predictive biomarkers, with candidate tools such as TCR repertoire metrics, tumor-reactive TIL frequency, and single-cell sequencing of pre-treatment biopsies requiring prospective validation before rational selection for this resource-intensive therapy becomes feasible (66, 69, 73, 82).

## 5 CAR-T cell therapy in lung cancer

### 5.1 CAR-T design principles and engineering evolution

CAR-T cells carry a synthetic chimeric antigen receptor that directs T cell cytotoxicity against surface antigens independently of MHC class I, enabling recognition of targets inaccessible to conventional T cells (83, 84). Second-generation CARs, the current clinical standard, incorporate a single co-stimulatory domain: CD28 drives rapid effector function, while 4-1BB promotes T cell memory and persistence (26, 83). Third-generation CARs incorporate two co-stimulatory domains simultaneously but have not demonstrated consistent clinical superiority over second-generation designs and remain less widely adopted (85). Fourth-generation ‘armored’ CARs additionally encode cytokine payloads such as IL-15, IL-7/CCL19, or inducible IL-12, activated upon tumor engagement to remodel the immunosuppressive microenvironment (85). Next-generation designs address two central problems: off-tumor toxicity and antigen escape. Logic-gated constructs use Boolean control to restrict killing: AND-gate designs require simultaneous recognition of two tumor antigens to activate cytotoxicity, while inhibitory NOT-gate CARs suppress killing upon recognition of a ‘self’ antigen expressed on normal tissue, both strategies sparing tissues that lack the full tumor-specific antigen combination, while dual-targeting CARs engage two antigens at once, preventing tumor escape through single-antigen loss. Alternative antigen-binding scaffolds such as nanobodies and DARPins, which are smaller and more structurally flexible than conventional antibody fragments, extend recognition to antigen configurations that standard CARs cannot access (86, 87).

These design choices are not equally consequential across diseases, and lung cancer selects among them sharply. Because the leading antigen candidates in this disease, discussed in Section 5.2, are expressed on normal lung epithelium rather than on a lineage the patient can survive losing (88), the logic-gated and dual-antigen architectures are better understood here as preconditions for an acceptable therapeutic window than as incremental refinements, and it is notable that the first complete response recorded in NSCLC came from a logic-gated construct rather than a conventional one (89). Because the suppressive environment described in Section 2 acts over weeks rather than hours, the co-stimulatory domain choice matters less for initial cytotoxicity than for whether the product persists long enough to matter, which favors 4-1BB-driven memory differentiation over CD28-driven rapid effector function (26, 83). Armored payloads address a microenvironment that is suppressive by default rather than merely indifferent (85). The design space therefore narrows considerably once lung-specific constraints are applied, although the programs currently in trials span both conventional and logic-gated architectures, and whether the narrowing is borne out will depend on results that are not yet available.

### 5.2 Antigen targets for CAR-T in lung cancer

Multiple surface antigens have been evaluated as CAR-T targets in lung cancer. EGFR is overexpressed in >60% of NSCLC tumors in certain populations; a Phase 1 trial (NCT03182816) of piggyBac-integrated EGFR CAR-T achieved one partial response and six stable disease outcomes in nine patients with an acceptable safety profile, while a follow-on study (NCT05060796) evaluates CXCR5-modified EGFR CAR-T to improve intratumoral traffic (88, 90, 91). Mesothelin, expressed in approximately 53% of lung adenocarcinomas, has been targeted via intrapleural delivery in malignant pleural mesothelioma (not NSCLC, included here as the most clinically advanced mesothelin CAR-T data in a thoracic malignancy), yielding a median OS of 23.9 months when combined with pembrolizumab (92) and a 64% ORR with disease control in all patients when CAR-T cells were engineered to co-secrete anti-PD-1 nanobodies upon IFN-γ activation, establishing proof of concept for intrinsic checkpoint blockade within the cellular product in a thoracic malignancy (93). B7-H3, broadly overexpressed across NSCLC and SCLC with limited normal tissue expression, is the target of a recently registered NCI-sponsored Phase 1 trial in ES-SCLC (NCT07509034) (94). DLL3-targeted AMG 119 was evaluated in a Phase 1 study in SCLC (95); updated results demonstrated preliminary antitumor activity, including one confirmed partial response among five patients (96). PSCA and MUC1 have demonstrated preclinical efficacy in NSCLC, including superiority of dual-CAR combination over monotherapy, with MUC1 CAR-T plus CRISPR PD-1 knockout having entered clinical testing (9, 88, 97). Additional targets including ROR1, HER2, CEA, and FAP remain in early investigation; PD-L1-directed programs encountered serious adverse events attributable to on-target/off-tumor toxicity on immune cells (9, 88, 98).

The pattern across these programs is more informative than any individual result. None of these targets reproduces the properties that made CD19 tractable, and the reasons are systematic rather than incidental. CD19 is expressed uniformly, at high surface density, and is confined to a lineage the patient can survive losing (99). No candidate in NSCLC or SCLC satisfies all three conditions simultaneously. EGFR and mesothelin are both expressed on normal lung epithelium, including alveolar type 1 and type 2 cells, ciliated cells and basal cells (88), so the therapeutic window in this disease is set by normal tissue tolerance rather than by tumor biology, and the serious on-target off-tumor events that ended PD-L1-directed programs illustrate what happens when that window is misjudged (9, 88, 98). Where expression is tumor-restricted, it is typically heterogeneous, and heterogeneity is not simply a matter of some cells being missed. Intermediate antigen density falls below the threshold required for productive CAR signaling, so antigen-low subclones are not only spared but are selected for under treatment pressure, while the engineered cells receiving subthreshold stimulation are driven toward exhaustion rather than sustained cytotoxicity (100, 101). Antigen heterogeneity in solid tumors is therefore a signaling problem as much as a coverage problem, and this is why strategies that lower the activation threshold, such as increased CAR signal strength or logic-gated dual-antigen recognition, are more likely to change outcomes than the continued search for a solid tumor equivalent of CD19.

### 5.3 Genome-editing approaches

Genome editing of T cells offers complementary strategies to improve CAR-T performance in lung cancer. The first-in-human Phase 1 trial of CRISPR-Cas9-edited T cells in refractory NSCLC (NCT02793856), disrupting PDCD1 (PD-1), confirmed clinical feasibility and safety, with no dose-limiting toxicities and with a median off-target mutation frequency of 0.05%, predominantly in intergenic and intronic regions, and no evidence of oncogenic transformation, alongside a median editing efficiency of 5.81% (102). Notably, this trial edited endogenous, nonreceptor-engineered T cells rather than a CAR-T or TCR-T product; its relevance here is as a safety and feasibility precedent for the editing infrastructure, not as clinical evidence for either engineered-receptor modality. More precise editing tools such as base editors can modify individual DNA letters without cutting the genome, enabling targeted changes such as disabling PD-1 surface expression on CAR-T cells so they resist being switched off by checkpoint signals. Separately, genome-wide CRISPR screens, where every gene in the T cell is systematically deleted and the effect on tumor killing is measured, have identified genes such as RASA2, SOCS1, CBLB, and TCEB2 that act as brakes on T cell activity, and removing them enhances CAR-T cytotoxicity and survival within the immunosuppressive tumor environment (103).

Whether these approaches are deployable in lung cancer is a separate question from whether they work in a model system, and three obstacles stand between the two. The first is efficiency: the clinical precedent in this disease achieved a median editing efficiency of 5.81%, sufficient to demonstrate feasibility but far below what a therapeutic multiplex-edited product would require, and each additional locus compounds the shortfall. The second is that these targets are brakes by function rather than by accident. RASA2 restrains antigen responsiveness and SOCS1 acts as a non-redundant intrinsic inhibitor of T cell activity, so deletion enhances potency precisely by removing a physiological constraint (103). That this can have consequences is not speculative: biallelic TET2 disruption combined with sustained BATF3 expression has been shown to drive antigen-independent proliferation in CAR-T cells, establishing that removing intrinsic brakes can uncouple proliferation from antigen engagement, an outcome standard potency assays are not designed to detect (103). The third is genomic integrity under multiplexing. Simultaneous double-strand breaks at several loci substantially increase the risk of chromosomal translocations and large structural variants, and detectable translocation frequencies have been reported in triple- and quadruple-edited T cell products, which is why genome-wide assessment and longitudinal post-infusion monitoring are increasingly expected for edited products (103). None of these obstacles is insurmountable, but together they explain why genome-edited CAR-T products remain preclinical in this disease while the underlying screens continue to generate candidate targets.

### 5.4 Engineering strategies to overcome solid tumor barriers

The barriers established in Section 2, hypoxic and adenosine-rich tissue, a suppressive myeloid compartment, and physical exclusion, define three sequential problems a CAR-T cell must solve: it must localize to the tumor, penetrate physical barriers, and survive once inside. Each has been addressed by dedicated engineering strategies (98). Chemokine receptor co-expression creates directed migratory gradients: CCR2b-modified CAR-T cells achieved a 12.5-fold greater intratumoral accumulation in preclinical models, and CXCR5 modification is being evaluated in the NCT05060796 EGFR CAR-T trial to exploit CXCL13 signaling at tertiary lymphoid structures in NSCLC (91, 104–106). For TME survival, membrane-bound IL-15 preserves a stem-like T cell phenotype with superior persistence (107); chimeric TGF-β receptors and SMAD7 knock-in convert suppressive TGF-β signaling into an activating signal (103, 108); A2AR disruption abrogates adenosinergic immunosuppression, relevant in EGFR- and KRAS-mutant NSCLC (103, 104); and constitutive anti-PD-1 nanobody secretion within the CAR-T product achieved a 64% ORR in malignant mesothelioma patients in a Phase 1 trial, demonstrating that checkpoint blockade embedded in the cellular product can overcome the immunosuppressive tumor microenvironment without systemic co-administration (93).

### 5.5 Current clinical landscape and key challenges

Published CAR-T trials in lung cancer have enrolled small numbers of patients across heterogeneous antigen targets and engineering platforms; a systematic review identified 21 registered trials and 5 publications reporting outcomes in 48 NSCLC and 5 SCLC patients, with a global ORR below 10%, against complete remission rates of 70–90% with CD19-directed CAR-T in B-cell acute lymphoblastic leukemia, and exceeding 50% in large B-cell lymphoma (96, 109, 110).

More recent single-arm trials suggest this ceiling may be shifting. A hypoxia-responsive CEA-directed CAR-T construct achieved a 47% objective response rate and an 87% disease control rate in 15 heavily pretreated NSCLC patients, with the benefit concentrated in patients with high tumor CEA expression (111). Separately, a logic-gated Tmod CAR-T construct (A2B694, EVEREST-2) has produced the first complete response to CAR-T therapy in a patient with metastatic NSCLC (89). Both results are drawn from small, single-arm, early-phase cohorts and await confirmation in larger series, but they indicate that newer-generation constructs are already exceeding the historical sub-10% benchmark.

The gap must be weighed on the mechanism of action over TCR-dependent modalities. CAR-T recognition is MHC-independent; therefore it retains activity against tumors that downregulate HLA class I to evade these other modalities. Several barriers converge to explain this gap: no NSCLC antigen equivalent to CD19 exists with uniformly high, tumor-restricted expression; intratumoral heterogeneity and intermediate antigen density cause subthreshold CAR activation, a signaling deficit that not only allows antigen-low tumor cells to escape recognition but also drives the CAR-T cells themselves toward anergy and exhaustion rather than sustained cytotoxicity (98–101); dense stroma, aberrant vasculature, and chemokine receptor mismatch limit intratumoral T cell accumulation; and the immunosuppressive milieu of TAMs, Tregs, MDSCs, TGF-β, and adenosine drives rapid functional exhaustion upon T cell entry (88, 112). Dual-antigen targeting strategies represent a direct mitigation against antigen escape.

### 5.6 Allogeneic “off-the-shelf” CAR-T

Autologous CAR-T manufacturing, requiring three to four weeks from apheresis to infusion, poses logistical barriers in lung cancer, where disease progression during production frequently renders patients ineligible for infusion, and T cells harvested from heavily pretreated patients often carry pre-existing exhaustion that limits product quality (113, 114). Allogeneic ‘off-the-shelf’ CAR-T products, derived from healthy donors and cryopreserved as standardized doses, can be delivered within days of enrollment; the principal engineering challenge is preventing graft-versus-host disease through TRAC locus disruption and host rejection through B2M knockout combined with HLA-E expression to evade NK cell surveillance (113, 114). Clinical proof of concept in solid tumors was provided by the TRAVERSE Phase 1 trial of ALLO-316, an allogeneic CD70 CAR-T in advanced clear cell renal cell carcinoma, a non-lung solid tumor, included here as proof-of-concept for the allogeneic manufacturing platform rather than as NSCLC- or SCLC-specific evidence, which achieved a 33% confirmed ORR in patients with high CD70 expression, with no GvHD events but a substantial toxicity burden including grade ≥3 adverse events in 84% of patients and three treatment-related deaths (115). Emerging platforms such as iPSC-derived T cells and γδ T cells offer manufacturing scalability and immune-compatibility advantages for further allogeneic development (113, 114).

## 6 TCR-engineered T cell therapy in lung cancer

### 6.1 Principles and advantages over CAR-T in solid tumors

TCR-T cells recognize peptides from intracellular proteins presented on MHC class I molecules, granting access to the full tumor proteome, including intracellular targets such as oncoproteins and mutant driver genes inaccessible to CAR-T cells, which are limited to surface antigens (116). This recognition mechanism enables high antigen sensitivity, with as few as 1–50 peptide-MHC complexes per cell sufficient to trigger cytolysis, below the threshold required for CAR-T activation, making TCR-T well suited to antigen-low solid tumors such as NSCLC (15, 116). TCR signaling through the native CD3 complex relies on near-physiological co-stimulatory engagement, which may favor memory T cell formation and sustained *in vivo* persistence compared with first-generation CAR constructs lacking co-stimulatory domains, though this advantage over modern 4-1BB- or CD28-containing CARs remains to be established in direct comparisons (116, 117). Manufacturing involves identifying tumor-reactive TCR sequences from patient TILs or peripheral blood, cloning the α and β chains into a viral vector, and introducing them into autologous T cells, with optimization steps to prevent mispairing with endogenous TCR chains (116, 118). In lung cancer this sensitivity advantage is purchased at a cost the CAR-T platform does not pay, since MHC restriction ties eligibility to HLA haplotype and antigen expression simultaneously (116). The compound effect of those two filters, rather than any deficiency in the receptor itself, is what has limited TCR-T programs in NSCLC to the small treated populations described in Section 9.5, where roughly 45% of screened patients carried the required HLA-A*02 haplotype but the combined haplotype and antigen match fell to about 4.5%, and under 1% were ultimately treated (25, 119).

### 6.2 Tumor-associated antigen-targeted TCR-T

TAA-targeted TCR-T therapy has focused primarily on cancer germline antigens, proteins normally restricted to germ cells but aberrantly re-expressed in tumors, such as MAGE family members and NY-ESO-1 (116). Early MAGE-A3 TCR-T trials were halted due to fatal cardiac and neurological toxicities from cross-reactivity with titin in cardiomyocytes and MAGE-A12 in the brain, respectively, establishing the need for thorough normal tissue safety profiling for all engineered TCRs (15, 116, 120). Critically, the standard preclinical safety panel used at the time, screening against a panel of normal human cell lines, did not detect the titin cross-reactivity, and two patients died of acute cardiac failure within five days of T cell infusion; *post hoc* analysis identified peptide scanning (systematic alanine-substitution mutagenesis of the target epitope) combined with more physiologically complex culture systems, including cardiomyocyte-containing assays, as approaches that could have flagged this cross-reactivity before clinical testing, and both are now recommended as standard preclinical screening steps for any affinity-enhanced TCR construct (120).

MAGE-A4-targeted therapy has proven safer: the SPEARHEAD-1 trial of afami-cel in synovial sarcoma (not a lung cancer trial, but the approved TCR-T reference class against which lung-specific programs are benchmarked) achieved a 39.4% ORR and led to FDA accelerated approval, the first for any TCR-T therapy (121). The related MAGE-A10 program in NSCLC was less encouraging, yielding only one partial response in 11 evaluable patients, reflecting the distinct immunological barriers of the lung tumor microenvironment (15, 116, 122). NY-ESO-1 TCR-T has shown a 47% average ORR among 107 patients across five clinical trials, with the strongest results in melanoma and synovial sarcoma, and additional activity demonstrated in myeloma (116, 123). All TAA-targeted TCR-T programs face two structural limitations: central tolerance requires affinity enhancement that risks off-tumor cross-reactivity, and HLA restriction means each product applies only to patients carrying the specific presenting allele, together narrowing the eligible NSCLC populations (119).

### 6.3 Neoantigen-specific TCR-T therapy

Tumor-specific mutational neoantigens, expressed exclusively on cancer cells, absent from normal tissues, and not subject to central tolerance, represent an immunologically superior class of TCR-T targets (124, 125). They fall into two categories: private neoantigens from patient-specific mutations requiring a personalized pipeline, and public neoantigens from recurrent hotspot mutations enabling standardized off-the-shelf TCR products (126). Proof of concept was demonstrated in a patient with metastatic pancreatic cancer who achieved a partial response with 72% tumor reduction following infusion of autologous T cells engineered to express two HLA-C08:02–restricted TCRs targeting mutant KRAS G12D, with engineered cells constituting more than 2% of circulating T cells at six months (116, 127). Subsequent work identified TCRs targeting the recurrent CTNNB1 S37F β-catenin mutation presented on HLA-A*02:01 and HLA-A*24:02, with engineered T cells eradicating established tumor xenografts in preclinical models and showing no reactivity against wild-type antigen, establishing this as a tractable shared neoantigen target across CTNNB1-mutated solid tumors (125, 126).

### 6.4 Clinical experience in NSCLC

Clinical TCR-T experience in NSCLC remains limited. The CRISPR-Cas9 multi-gene editing trial (NCT02793856) established that genomic editing of T cells is safe and feasible in lung cancer patients, with a median off-target mutation frequency of 0.05% (range 0–0.25%) and no dose-limiting toxicities (102, 116); although that trial targeted PDCD1 rather than TCR loci, it demonstrated the clinical viability of the editing infrastructure that TCR knock-in approaches now depend on. The leading transgenic TCR program in NSCLC, letetresgene autoleucel (GSK3377794), targets NY-ESO-1/LAGE-1A on HLA-A02:01, HLA-A02:05, or HLA-A02:06 but enrolled only 18 treated patients from more than 2,500 screened, an eligibility rate below 1% from compound HLA and antigen positivity constraints (119, 128). Results were limited, with one partial response in five monotherapy patients and no objective responses in 13 multi-arm patients, attributed to T cell trafficking failure, exhaustion from prior immunotherapy, antigen presentation defects, and potential HLA loss of heterozygosity in progressing lesions (119).

### 6.5 Challenges and future directions for TCR-T

MHC class I downregulation, through B2M mutation, epigenetic silencing, or defective antigen processing, is the principal TCR-T-specific vulnerability, rendering tumor cells invisible to any MHC-dependent recognition strategy while leaving CAR-T and BiTE activity intact (25, 119). HLA restriction is the central scalability barrier: every TCR recognizes a specific peptide only in the context of a defined allele, compounding with antigen prevalence to produce the sub-1% eligibility rate seen in letetresgene autoleucel NSCLC trials (119, 128). Identifying TCRs for the same shared neoantigen across multiple common HLA alleles, as demonstrated for KRAS G12D and CTNNB1 S37F, and integrating HLA typing into standard next-generation sequencing panels at diagnosis are two complementary strategies to expand eligible populations (119, 126, 127). For personalized neoantigen TCR-T, AI-assisted prediction tools and CRISPR-based TCR knock-in are shortening the manufacturing timeline, with early feasibility studies in 16 solid tumor patients confirming safety and tumor homing of personalized TCR-T products (116, 124, 125).

## 7 Toxicity management across T cell-redirected modalities

### 7.1 CRS and ICANS: grading and management

Cytokine release syndrome (CRS) is the predominant acute toxicity across T cell-redirected therapies such as BiTEs, CAR-T, and TIL, arising from rapid systemic inflammation triggered by T cell activation and driven primarily by IL-6, IL-1, IFN-γ, and TNF-α (129). The ASTCT consensus grading system standardizes CRS severity on a four-point scale anchored by fever, hemodynamic, and respiratory parameters, enabling consistent cross-trial safety assessment (130). Management follows a graded-response algorithm: grade 1 requires supportive care; grade 2 warrants tocilizumab (IL-6 receptor blockade) plus IV hydration; grade 3–4 requires tocilizumab combined with systemic corticosteroids and ICU-level monitoring, with anakinra as a salvage option in refractory cases (129, 131–134). ICANS, the second major class-effect toxicity, arises from cytokine-mediated blood-brain barrier disruption, manifesting as encephalopathy, confusion, and in severe cases seizures or cerebral edema; it is graded using the ICE score and managed with supportive care at grade 1; corticosteroids (dexamethasone or high-dose methylprednisolone) are initiated from grade 2 onward, with escalating doses at higher grades; tocilizumab is ineffective for CNS toxicity (130, 131, 135).

### 7.2 Modality-specific toxicity considerations

For tarlatamab specifically, CRS occurs in ~50% of patients but is predominantly grade 1–2, front-loaded in early cycles, and rapidly self-resolving; a mandatory step-up dosing strategy (1 mg priming before 10 mg target dose) mitigates severity, tocilizumab was required in only 11% of CRS events, and the overall profile supports outpatient administration in experienced centers (45, 136–138). Beyond CRS and ICANS, each modality carries additional considerations: allogeneic CAR-T requires vigilance for GvHD, mitigated by TRAC/B2M CRISPR engineering (114); TCR-T programs targeting shared tumor-associated antigens carry on-target/off-tumor toxicity risk, illustrated by fatal cardiac toxicity from MAGE-A3 TCR cross-reactivity with titin (118, 129); and TIL therapy produces toxicity from the lymphodepletion regimen and high-dose IL-2, including hypoxia, febrile neutropenia, and prolonged cytopenias, requiring close hematological monitoring and growth factor support (65, 81, 131, 135).

## 8 Resistance mechanisms and strategies to overcome them

Despite clinical activity across all T cell-redirected modalities, primary and acquired resistance remains a fundamental barrier. Resistance mechanisms can be organized into three interconnected categories such as antigen-level resistance, T cell-intrinsic functional failure, and TME-mediated immunosuppression, each demanding distinct counter-strategies (58).

### 8.1 Antigen-level resistance

The central resistance mechanism shared across all antigen-targeted T cell therapies is loss or downregulation of the target antigen. In B-cell malignancies, antigen-negative clonal escape is a major mechanism of CAR-T relapse in B-cell malignancies, with CD19-negative escape rates ranging from 7–25% across trials depending on follow-up duration and patient population; in solid tumors including NSCLC, the problem is compounded by pre-existing intratumoral heterogeneity, with antigen-low subclones present before therapy begins and selectively expanded under sustained immune pressure (59, 99). For tarlatamab and DLL3-targeted therapies, DLL3 downregulation driven by NOTCH pathway alterations under treatment pressure is an emerging resistance mechanism; in parallel, IFN-γ released by activated T cells drives PD-L1 upregulation on tumor cells, providing a secondary adaptive evasion mechanism (58, 59). Dual-targeting CAR constructs, bispecific architectures, and logic-gated constructs requiring co-expression of two non-overlapping antigens are direct mitigation strategies, alongside approaches to lower the antigen-density threshold required for T cell activation (86, 99).

### 8.2 T cell-intrinsic resistance

The exhaustion program described in Section 2.2 is not only a property of the endogenous T cell compartment but a failure mode of the engineered product itself, and its causes are distinct. In CAR-T products, exhaustion is accelerated by tonic CAR signaling and manufacturing protocols that preferentially yield terminally differentiated effectors rather than the progenitor-like memory cells capable of self-renewal (103). Selecting 4-1BB over CD28 co-stimulation promotes central memory differentiation and longer persistence; CRISPR knockout of exhaustion drivers such as TOX, NR4A1, and PD-1 reduces susceptibility; membrane-bound IL-15 enriches stem-like T cell phenotypes with cytokine-independent persistence; and FOXO1 overexpression reprograms products toward memory phenotypes before infusion (86, 103, 107, 139).

### 8.3 TME-mediated immunosuppression

Even with adequate antigen expression and a functionally competent T cell product, the lung TME erects multiple barriers to therapy. Dense stroma, dysfunctional tumor vasculature, and a chemokine receptor mismatch between manufactured T cells and the tumor’s secretory profile collectively impede intratumoral accumulation (17, 104). Within the tumor, TGF-β suppresses effector function (103); MHC class I downregulation eliminates TCR-dependent recognition (25); while CAR-T and BiTE recognition, being surface antigen directed and MHC-independent, remains intact under this evasion route; and Tregs and MDSCs establish a broadly immunosuppressive niche that impairs T cell function through cytokine, metabolic, and checkpoint-mediated mechanisms (17).

These barriers do not weigh equally across modalities, and the differences are practically consequential. Stromal exclusion and impaired trafficking are decisive for adoptive products, which must physically reach the tumor after infusion, but weigh differently on T cell engagers, which act on T cells already resident in the tissue and are limited instead by how few of those cells remain functional (17, 104, 106). MHC class I downregulation eliminates recognition by TIL and TCR-T therapy while leaving CAR-T and BiTE activity intact, which makes antigen presentation status a modality-determining rather than merely prognostic variable (25). The adenosinergic and myeloid suppressive axes affect all four (21), but their consequences differ in timing: an engager acts on a T cell pool that may already be exhausted at the moment of dosing, whereas an adoptive product delivers cells that are functional on infusion and become progressively suppressed thereafter, which is why persistence measures matter for one and target density matters more for the other. The engineering responses to these barriers are discussed in Section 5.4.

## 9 Combination strategies and future directions

The barriers confronting T cell-redirected therapy in lung cancer, such as an immunosuppressive tumor microenvironment, antigen heterogeneity, T cell exhaustion, and manufacturing constraints, are unlikely to yield to any single modality in isolation. Converging strategies that address multiple axes simultaneously represent the clearest path toward durable clinical benefit.

### 9.1 T cell-redirected therapy combined with checkpoint inhibitors

BiTE-induced T cell engagement simultaneously upregulates PD-1 and other inhibitory receptors, creating an exhaustion feedback loop that blunts response durability, making concurrent checkpoint inhibitor co-administration mechanistically rational across all T cell-redirected modalities (10). For tarlatamab in ES-SCLC, the DeLLphi-303 trial of tarlatamab combined with a PD-L1 inhibitor as first-line maintenance demonstrated a manageable safety profile and a median OS of 25.3 months from the start of tarlatamab maintenance, in a context where historical PD-L1 inhibitor maintenance data show 12–13 months OS from start of first-line therapy; however, randomised comparison data are awaited (47, 48). In NSCLC, the IOV-COM-202 trial of lifileucel plus pembrolizumab in ICI-naïve advanced NSCLC achieved a 64.3% ORR in the EGFR-wild-type subgroup, establishing cooperative activity between polyclonal TIL antigen broadening and PD-1 disinhibition (65, 81). For CAR-T, embedding anti-PD-1 nanobody secretion within the cell product itself achieved a 64% ORR in a Phase 1 mesothelioma trial, providing proof of concept that intrinsic checkpoint blockade delivered locally at the tumor interface may be more effective than systemic co-administration, a strategy directly applicable to pleural and thoracic T cell-redirected programs (93).

### 9.2 Combining T cell-redirected therapy with antibody-drug conjugates

ADCs and T cell-redirected therapies act as synergistic partners: ADC-mediated tumor killing generates immunogenic cell death that primes T cells and augments infiltration, while BiTE- or CAR-T-induced IFN-γ can upregulate ADC target expression on surviving tumor cells, creating reciprocal immunological enhancement (36). DLL3 and B7-H3 are the two leading convergence nodes in lung cancer (140). Ifinatamab deruxtecan (I-DXd), a B7-H3-directed ADC, demonstrated a 48.2% ORR in 137 previously treated ES-SCLC patients in the IDeate-Lung01 Phase 2 trial, informing the ongoing Phase 3 IDeate-Lung02 study comparing I-DXd versus chemotherapy in second-line SCLC, and the Phase 1b/2 IDeate-Lung03 study combining I-DXd with atezolizumab as first-line therapy (141–143). The recently registered NCI Phase 1 B7-H3 CAR-T trial in ES-SCLC creates an opportunity to explore concurrent or sequential ADC and CAR-T approaches targeting the same antigen (94). In NSCLC, HER3-DXd and datopotamab deruxtecan represent ADC platforms that could synergize with emerging HER3- or TROP2-directed T cell-redirected programs (36).

### 9.3 Next-generation CAR-T and TCR-T engineering platforms

Two transformative engineering approaches aim to eliminate the manufacturing burden of current CAR-T therapy. *In vivo* CAR-T generation, delivering viral vectors or lipid nanoparticles encoding the CAR directly into the patient, transduces T cells *in situ* without apheresis or ex vivo expansion; mRNA-based transient CAR expression reduces exhaustion risk from chronic tonic signaling and enables repeated dosing (103). Synthetic biology platforms are enabling more precise tumor-targeting logic: SUPRA-CAR systems allow modular antigen retargeting by swapping a soluble adaptor without re-engineering the cell; logic-gated AND-gate circuits require co-expression of two antigens for T cell activation, reducing on-target/off-tumor toxicity; and bispecific dual-targeting constructs simultaneously engage two non-overlapping antigens to prevent single-antigen escape, all directly applicable to the heterogeneous NSCLC antigen landscape (86). For TCR-T, deep learning neoantigen prediction tools and single-cell TCR sequencing with peptide-MHC multimers are transforming the identification and prioritization pipeline, shortening the path from tumor sequencing to a therapeutic product (125).

### 9.4 Allogeneic, NK cell, and innate T cell platforms

Allogeneic “off-the-shelf” CAR-T products, manufactured from healthy donors with TRAC/B2M/HLA-E engineering to prevent GvHD and host rejection, eliminate the manufacturing delays and T cell fitness problems of autologous approaches, with early solid tumor clinical data supporting feasibility (113, 114). CAR-NK cells offer distinct advantages: they kill through multiple MHC-independent mechanisms, lack an endogenous αβ TCR, eliminating GvHD risk without additional engineering, produce less severe CRS, and can target MHC-low or antigen-heterogeneous tumor populations that escape CAR-T; preclinical studies targeting EGFR, EpCAM, and HER2 in lung cancer models have confirmed potent antitumor activity (144). Invariant NKT (iNKT) cells recognize lipid antigens via the monomorphic CD1d molecule independently of MHC class I, bypassing antigen presentation defects entirely; upon activation they deploy broad effector programs such as direct cytotoxicity, NK cell transactivation, and pro-inflammatory cytokine secretion, remodeling the immunosuppressive TME and positioning CAR-iNKT constructs as potential combination partners for BiTEs and TIL therapies in lung cancer (145).

### 9.5 Biomarker-driven patient selection

The heterogeneity of lung cancer biology across patients and the mechanistically distinct requirements of each T cell-redirected modality make biomarker-driven patient selection essential for maximizing therapeutic benefit (59). Without prospective enrichment strategies, the proportion of unselected patients who derive meaningful benefit will remain insufficient to drive broad adoption, as the letetresgene autoleucel NSCLC experience, where less than 1% of screened patients were ultimately treated, demonstrated (119).

For tarlatamab and other DLL3-directed T cell engagers in SCLC, patients with high DLL3 expression are considered the best candidates for treatment, but there’s no agreed-upon DLL3 cutoff that reliably predicts who will respond. In the pivotal DeLLphi-300 trial, patients responded whether their tumors tested DLL3-positive or DLL3-negative, meaning DLL3 test results didn’t actually separate likely responders from non-responders. Since about 96% of tumors test positive for DLL3, testing mainly confirms that a patient qualifies for treatment rather than predicting response. The molecular subtype architecture of SCLC, including SCLC-A (ASCL1-high, highest DLL3 expression), SCLC-N (NEUROD1), SCLC-P (POU2F3), and SCLC-I (inflamed immune signature), provides a complementary framework: SCLC-A tumors are the natural candidate population for DLL3-directed BiTEs and ADCs, while SCLC-I may benefit most from checkpoint inhibitor combinations and adoptive T cell approaches (59).

This mapping is a snapshot rather than a fixed property, since the subtype architecture is plastic under therapeutic pressure and the ASCL1-high to NEUROD1-high transition that accompanies DLL3 downregulation, described in Section 3.5, means a patient selected on subtype at baseline may not retain that subtype at progression (58, 59). Subtype is therefore a criterion for initial selection whose predictive value decays over treatment, which argues for serial rather than single-timepoint molecular assessment in trials of DLL3-directed therapy.

For TIL therapy in NSCLC, TCR repertoire clonality and the ratio of tumor-reactive to non-tumor-reactive TILs in the manufacturing biopsy are emerging as candidate selection metrics; Han et al. developed the TCR-based immunotherapy response index, a measure of TCR clone sharing between tumor-infiltrating and circulating PD-1+CD8+ T cells, as a classifier of ICI response in NSCLC, providing proof-of-concept that intratumoral TCR repertoire characteristics are clinically informative (146). This index measures how much overlap exists between the T cell receptors found in the tumor and those found in circulating PD-1+CD8+ T cells in the blood. In the original study, a cutoff score of 1.91 discriminated responders from nonresponders; whether analogous metrics can predict TIL therapy outcomes awaits dedicated prospective evaluation. None of these metrics have been prospectively validated or incorporated into TIL trial eligibility criteria; current NSCLC TIL trials enroll based on tumor accessibility and performance status rather than immune biomarker fitness, and prospective co-enrollment of manufacturing biopsy sequencing data within ongoing Phase 2 studies represents the nearest path to generating the validation evidence needed before biomarker-guided TIL selection becomes feasible.

For CAR-T and TCR-T therapies, MHC class I expression status is a non-negotiable eligibility filter, since MHC class I downregulation renders tumors invisible to TCR-dependent approaches while leaving CAR-T and BiTE sensitivity intact. The magnitude of this bottleneck is illustrated by the letetresgene autoleucel NSCLC screening funnel: approximately 45% of screened patients were HLA-A*02–positive, but NY-ESO-1 and LAGE-1a tumor antigen positivity was present in only 12% and 4% of tested patients respectively, yielding a combined HLA-and-antigen match in roughly 4.5% of screened patients before further clinical eligibility criteria reduced the treated population to under 1% (25, 119). Tumor mutational burden, neoantigen load, and HLA allelic diversity inform the probability that a sufficient neoantigen TCR-T target exists for personalized programs (124, 125). Circulating tumor DNA and minimal residual disease monitoring by ultra-deep sequencing provide dynamic non-invasive response tracking applicable across all modalities, with early ctDNA decline identifying responders and rising ctDNA signaling emerging resistance before radiographic progression (147). Integrating antigen expression, molecular subtype, TCR repertoire, HLA status, and ctDNA kinetics into adaptive trial designs will be essential for translating the biological promise of T cell-redirected therapy into durable clinical benefit across the diverse molecular landscape of lung cancer.

### 9.6 Economic and logistical barriers to implementation

Manufacturing timeline and cost together determine clinical feasibility across all four modalities. Tarlatamab and other BiTEs are off-the-shelf biologics needing no patient-specific manufacturing, so treatment can start immediately. TIL therapy requires a 22-day GMP process within an overall 4–6 week vein-to-vein timeline, and autologous CAR-T and TCR-T need a comparable 3–4 week apheresis-to-infusion timeline; both frequently require bridging therapy since patients can progress, or manufacturing can fail, during this window (66, 72, 73, 113, 114). Cost varies just as widely. Commercially manufactured lifileucel exceeds $500,000 per treatment, while hospital-based in-house manufacturing has been reported at approximately $55,000 per patient (66, 72). No lung cancer-specific CAR-T or TCR-T product is approved yet, but reference-class products suggest the range: CD19-directed CAR-T list prices run $373,000 to $633,000, and the approved TCR-T product afamitresgene autoleucel costs $727,000 per dose, figures that likely understate total cost of care once lymphodepletion and toxicity management are included. Tarlatamab, by contrast, is dosed biweekly at roughly $30,000 to $31,500 per cycle rather than as a one-time product, a different cost structure that complicates direct comparison with autologous cell therapies.

Allogeneic “off-the-shelf” platforms and CAR-NK cells have both been proposed to ease these cost and timeline barriers, but neither is a mature solution yet. Allogeneic CAR-T, manufactured in bulk from healthy donors and cryopreserved for immediate use, should eventually cost less than autologous manufacturing at industrial scale, though current products remain expensive and donor-specific anti-HLA antibodies can block redosing (113, 114). CAR-NK cells offer a parallel off-the-shelf approach with a better toxicity profile and no need for GvHD-preventing engineering, but face their own barriers: limited persistence and poor tumor trafficking reduce durable activity.

## 10 Discussion and conclusions

The four T cell-redirected modalities: BiTEs, TIL therapy, CAR-T, and TCR-T differ substantiallyin clinical maturity and best-positioned tumor type (**Tables1A**, **B**). Tarlatamab is the only modality with FDA traditional approval in lung cancer, establishing second-line ES-SCLC as the first approved T cell-redirected indication in thoracic oncology; its off-the-shelf availability and intracranial activity are strengths no autologous product currently matches, though the DLL3 target is restricted to neuroendocrine-lineage tumors and no pan-NSCLC equivalent has yet emerged (37, 43, 54, 55). TIL therapy is the leading adoptive cell therapy in NSCLC, with maturing Phase 2 efficacy data in both anti-PD-1-resistant and ICI-naïve settings (65, 72). CAR-T and TCR-T remain in early proof-of-concept phases, generating safety data and engineering signals that will guide next-generation product iterations (96, 119).

**Table 1a:** Mechanism of action, lung cancer activity, and clinical trial data.

Modality	Mechanism of action	Lung cancer activity	Clinical trial data
BiTE (tarlatamab)	Simultaneously bind tumor-associated antigen and CD3 complex on T cells; bridge T cells and tumor cells to form cytolytic synapse; MHC-independent (27)	>80% of SCLC tumors express DLL3 with minimal expression on normal tissues (31–35)	Phase 2 DeLLphi-301: 40% ORR at 10 mg dose in previously treated ES-SCLC (37, 40, 41) | Brain metastases: 45.3% ORR vs 32.6% without (40, 41), | Phase 3 DeLLphi-304: median OS 13.6 vs 8.3 months (HR 0.60) (43, 44)
TIL (lifileucel)	Harvest tumor-infiltrating lymphocytes; expand ex vivo with IL-2 and anti-CD3; reinfuse with non-myeloablative lymphodepletion + high-dose IL-2. MHC-dependent (36, 66, 71–73)	NSCLC lead candidate | First lung cancer trial: 3 of 13 evaluable NSCLC patients (23% ORR); 2 with complete responses ongoing at 1.5 years (64)	IOV-LUN-202 (non-squamous NSCLC): 25.6% ORR, 71.8% DCR, median DOR not reached at 25.4 months (65) | IOV-COM-202 + Pembrolizumab (ICI-naïve): 64.3% ORR EGFR-wild-type subgroup; 54.5% ORR in PD-L1-negative tumors (65, 81)
CAR-T	Engineer T cells ex vivo with synthetic CAR expressing antigen-binding domain + co-stimulation; second-generation with CD28 or 4-1BB; fourth-generation with IL-12/IL-15 payloads. MHC-independent (83–85)	Lung-specific targets: EGFR in >60% NSCLC (88, 90, 91), B7-H3 overexpressed in NSCLC (94), DLL3 in SCLC (95, 96)	Systematic review: 21 registered trials, 5 publications, 48 NSCLC + 5 SCLC patients (96, 109, 110) | EGFR CAR-T (NCT03182816): 1 partial response + 6 stable disease in 9 NSCLC patients (90, 91) | B7-H3 CAR-T: Phase 1 registered in ES-SCLC (NCT07509034) (94) | DLL3 AMG 119: 1 confirmed PR in 5 SCLC patients (95, 96)
TCR-T	Engineer T cells with tumor-reactive TCRs recognizing intracellular antigens via peptide-MHC class I presentation; HLA-dependent (116, 118) | Can target patient-specific neoantigens (125)	NY-ESO-1 TCR-T: 47% average ORR across 5 clinical trials (107 total patients (123) | NSCLC MAGE-A10: 1 partial response in 11 evaluable patients (122)	Letetresgene autoleucel (NY-ESO-1/LAGE-1A): <1% eligibility rate in NSCLC (<18 from >2500 screened) (119, 128) | Monotherapy: 1 PR in 5 patients; multi-arm: 0 objective responses in 13 patients (119) | CRISPR-Cas9 PDCD1 knockout (NCT02793856): safe in NSCLC patients; 0.05% off-target mutation frequency (102)

**Table 1b:** Key limitations, clinical stage, and manufacturing timeline.

Modality	Key Limitations	Clinical stage	Manufacturing timeline
BiTE (tarlatamab)	SCLC-restricted, no pan-NSCLC equivalent identified (40, 41) | Antigen loss via NOTCH pathway; PD-L1 upregulation on tumor cells (58, 59) | Real-world: CRS 72.7%, ICANS 40.9% (46)	FDA traditional approval Nov 2025 ES-SCLC (43–45)	Off the shelf, no patient-specific manufacturing
TIL (lifileucel)	4-6 week vein-to-vein manufacturing timeline (66, 72) | Cost >$500,000 commercial; ~$55,000 in-house (66, 72) | Requires patient fitness for non-myeloablative lymphodepletion and IL-2 (65, 79–81) | Majority of infused cells are non-tumor-reactive (63, 72)	Phase 2 (NSCLC) (78, 80) FDA approval melanoma (Feb 2024) (75, 76)	22-day GMP process; 4-6 week vein-to-vein (72, 73)
CAR-T	No uniformly expressed pan-NSCLC target (unlike CD19 in hematologic cancers) (54, 55, 99) | Tumor heterogeneity causes subthreshold CAR activation (98, 99) | Dense stroma and dysfunctional vasculature limit intratumoral accumulation (17, 88, 112) | Global ORR <10% in lung cancer (96, 109, 110)	Phase 1/2 Early proof-of- concept (96, 109, 110)	Autologous: 3-4 week apheresis-to-infusion; allogeneic: days (113, 114)
TCR-T	HLA class I restriction limits eligibility to <1% NSCLC population (119, 128) | MHC-I downregulation renders tumors invisible to TCR-dependent recognition (25, 119) | T cell exhaustion from prior ICI therapy (119) | MAGE-A3 program halted due to fatal cardiac/neurological toxicity from titin cross-reactivity (120)	Phase 1/2 Limited NSCLC data (119, 128)	Not defined, presumed to be very similar to CAR-T autologous timeline (116)

T cell-redirected therapy must be integrated into a landscape already shaped by checkpoint inhibitors, targeted therapies, and ADCs. For NSCLC without driver alterations, first-line pembrolizumab and chemoimmunotherapy remain the standard, with a 5-year OS of approximately 31.9% in PD-L1-high patients, underscoring the room for improvement these modalities aim to fill (6, 148). For ES-SCLC, Platinum-Etoposide-ICI remains the first-line standard with median OS of 12–15 months and nearly universal relapse; ongoing trials such as DeLLphi-305 are evaluating whether adding T cell-redirected therapy to this platform can improve on these outcomes, but the relapsed setting remains acutely underserved (19, 149). Tarlatamab has already established itself as the preferred second-line ES-SCLC option following the Phase 3 DeLLphi-304 superiority data versus chemotherapy, and first-line maintenance with tarlatamab plus atezolizumab represents its next likely algorithmic step pending Phase 3 validation (43, 44, 47, 48). I-DXd is advancing through IDeate-Lung02 as a potential alternative second-line SCLC option, with the prospect of future subtype-based sequencing between BiTE and ADC approaches (142). For NSCLC, TIL therapy is best positioned as a post-ICI option; CAR-T and TCR-T remain investigational in biomarker-selected trial settings (150).

Several questions remain unresolved. The optimal sequencing and combination of T cell-redirected therapies with ICIs, chemotherapy, and ADCs has not been systematically defined, and the immunological consequences of prior ICI therapy, including HLA loss, antigen presentation defects, and pre-established T cell exhaustion, are poorly characterized in the combination setting (11, 150). Predictive biomarkers remain the primary unmet need: without validated markers to identify likely responders, the therapeutic index of each modality will remain insufficient for broad adoption, as illustrated by the letetresgene autoleucel NSCLC experience where less than 1% of screened patients were treated. Prospective validation of DLL3 expression and SCLC molecular subtype for tarlatamab, TCR clonality for TIL therapy, and HLA class I status with antigen density for CAR-T and TCR-T programs is needed (11, 59). Manufacturing cost and accessibility, with autologous products exceeding hundreds of thousands of dollars per treatment and requiring specialized GMP infrastructure, constitute systemic equity barriers that allogeneic, CAR-NK, and *in vivo* CAR-T platforms must address to achieve population-scale deployment (11, 150).

The field’s most instructive lessons have come from its clearest failures. Rovalpituzumab tesirine demonstrated that a validated target can still fail in the clinic when the delivery platform carries its own narrow therapeutic index (section 3.1). The fatal MAGE-A3 TCR-T toxicities showed that affinity enhancement, undertaken to compensate for native TCRs’ low sensitivity, can introduce cross-reactivities invisible to conventional preclinical panels, now requiring the peptide-scanning and complex-culture screening now recommended for engineered TCRs (section 6.2). The sub-1% eligibility rate in the letetresgene autoleucel NSCLC program illustrates that biological rationale alone does not guarantee clinical reach when compound HLA and antigen-positivity requirements are stacked sequentially (section 9.5). These three programs argue that rigorous, mechanism-specific analysis of discontinued or underperforming trials is as valuable to the field’s progress as its clinical successes, and each has directly shaped the T cell engager, TCR-T safety-screening, and biomarker-selection strategies discussed throughout this review.

The near-term question for this field is not whether T cell redirection can work in lung cancer, which tarlatamab has settled for ES-SCLC (43, 44), but where each modality belongs in a treatment sequence that is already crowded. In oncogene-driven NSCLC the practical question is what happens after targeted therapy fails, and whether redirected T cell approaches are best deployed at that point or earlier, when disease burden is lower and the T cell compartment less compromised by successive lines of treatment. The interaction with prior EGFR-directed and KRAS G12C-directed therapy, including its effect on antigen presentation and on the adenosinergic axis enriched in these tumors (21), has not been systematically characterized. In ES-SCLC the question is whether tarlatamab moves earlier into first-line maintenance, as DeLLphi-305 is evaluating, and whether molecular subtype can direct patients between engager and conjugate approaches at relapse (142, 149). In NSCLC without a driver alteration, TIL therapy is positioned after checkpoint failure while CAR-T and TCR-T remain investigational (150), and they will stay there until either an antigen with adequate density and tumor restriction is identified or the activation threshold is engineered low enough that the antigens already available become sufficient. That last problem, more than manufacturing or cost, is what currently separates lung cancer from the hematological malignancies where these therapies succeeded.
